# Supplementary material for: The Effect of an Attenuated Live Vaccine against Salmonid Rickettsial Septicemia in Atlantic Salmon (Salmo salar) Is Highly Dependent on Water Temperature during Immunization
Source: Vaccines (Basel). 2024 Apr 15;12(4):416. doi: 10.3390/vaccines12040416 (PMC11053689; doi:10.3390/vaccines12040416)
Supplement: Supplementary file 1 [file vaccines-12-00416-s001.zip › vaccines-2946089-supplementary.pdf]

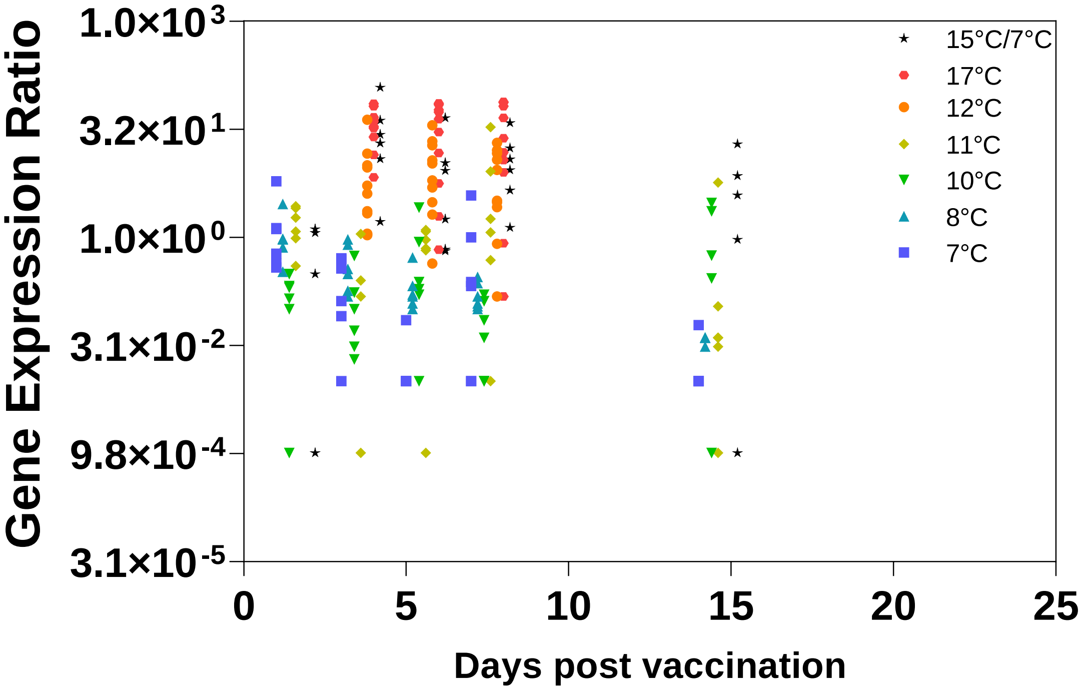

Figure S1. Normalized expression of *P. salmonis* RNA in livers from fish vaccinated and immunized at different temperatures. The figure is based on the Ct-values presented in Figure 5a normalized against corresponding Ct-values from the internal reference gene Elongation factor 1a from the same sample using the method outlined by Pfaffl et al 2001.
